# An Improved Multitask Learning Model with Matching Network and Its Application in Traditional Chinese Medicine Syndrome Recommendation

**DOI:** 10.1155/2022/4072563

**Published:** 2022-04-26

**Authors:** Yingshuai Wang, Jing-Han Xu, Meng Zhang, Dezheng Zhang, Aziguli Wulamu

**Affiliations:** ^1^Department of Computer, School of Computer and Communication Engineering, University of Science and Technology Beijing (USTB), Beijing 100083, China; ^2^Beijing Key Laboratory of Knowledge Engineering for Materials Science, Beijing 100083, China; ^3^Institution of Shenzhen Hospital, Guangzhou University of Chinese Medicine (Futian), Shenzhen 518000, Guangdong, China

## Abstract

Multitask learning (MTL) is an open and challenging problem in various real-world applications, such as recommendation systems, natural language processing, and computer vision. The typical way of conducting multitask learning is establishing some global parameter sharing mechanism among all tasks or assigning each task an individual set of parameters with cross-connections between tasks. However, for most existing approaches, the raw features are abstracted step by step, semantic information is mined from input space, and matching relation features are not introduced into the model. To solve the above problems, we propose a novel MMOE-match network to model the matches between medical cases and syndrome elements and introduce the recommendation algorithm into traditional Chinese medicine (TCM) study. Accurate medical record recommendation is significant for intelligent medical treatment. Ranking algorithms can be introduced in multi-TCM scenarios, such as syndrome element recommendation, symptom recommendation, and drug prescription recommendation. The recommendation system includes two main stages: recalling and ranking. The core of recalling and ranking is a two-tower matching network and multitask learning. MMOE-match combines the advantages of recalling and ranking model to design a new network. Furtherly, we try to take the matching network output as the input of multitask learning and compare the matching features designed by the manual. The data show that our model can bring significant positive benefits.

## 1. Introduction

Mixed negative sampling (MNS) [1] applies two-tower neural networks to improve retrieval quality in large-scale recommendation systems. The selection bias of implicit user feedback is solved via using the mixture of batch and uniformly sampled negatives. A neighbor similarity loss with a multichannel matching called Graph-DR framework [2] is proposed to improve both accuracy and diversity. YouTube general matching neural network [3] is applied to recall and sort in different stages, and joint training is proposed to solve the age exposure bias problem. A dual augmented two-tower model [4] is proposed, which employs adaptive-mimic mechanism (AMM) and category alignment loss (CAL) to model the information interaction between two towers and get better embedding for the imbalanced category. A deep structured semantic model (DSSM) [5] employs the click-through data to optimize the parameters that directly target the goal of ranking. Furthermore, DSSM extends the linear semantic structure to nonlinear using multiple layer perceptron, which can capture more sophisticated semantics. The adversarial two-tower neural network (ATNN) [6] model is applied to CTR prediction, which introduces an adversarial network to a two-tower network and extends it to multitask learning. Sampling bias-corrected neural (SBCN) [7] is proposed for estimating the item frequency of streaming data, which can be adaptive to data distribution and can reduce the sampling bias of in-batch items. A deep learning recommendation model (DLRM) [8] is proposed, which can mitigate memory constraints via utilizing model parallelism on the embedding tables. An internal and contextual attention network (ICAN) [9] is proposed, which combines the feature field interactions among multichannel with the channel-specific contextual attention in the matching module. An adversarial mixture of experts (ADMOE) [10] is proposed, which introduces adversarial regularization among the expert outputs and uses soft gating constraints of category hierarchy. Multitask adversarial active learning (MTAAL) [11] is proposed for medical named entity recognition and normalization, which can keep the performance of multitask learning and active learning via task discriminator and diversity discriminator. A hierarchical model with micro- and macro-behaviour (*HM*^3^) [12] is proposed, which utilizes multitask learning and applies the abundant supervisory labels from micro- and macro-behaviours to predict conversion rate (CVR) in a unified framework. Gating-enhanced multitask neural network (Gem-NN) [13] is proposed to predict CTR in a coarse-to-fine manner, which allows parameter sharing from upper-level tasks to lower-level tasks and introduces a gating mechanism between embedding layers and MLP. The multiple-level sparse sharing model (MSSM) [14] is proposed to represent feature flexibly and share information among tasks efficiently, which include a field-level sparse connection module (FSCM) and a cell-level sparse sharing module (CSSM). Affect dimensions in detecting emotions employing multitask learning [15] are proposed, which jointly trains multilabel emotion classifier and multidimensional emotion regressor via using the interrelatedness of tasks. An adaptive information transfer multitask (AITM) framework [16] is proposed, which uses the adaptive information transfer (AIT) module to construct the sequential dependence among audience multistep conversions. The sequential deep matching (SDM) model considering the users' dynamic evolution preference [17] is proposed, which can capture users' interests via combining long-term behaviours and short-term sessions.

In particular, our main contributions to this study can be summarized by the following four aspects:

1. We propose a novel two-tower match network for multitask learning, which can better learn ID embedding via auxiliary loss.
2. On the basis of a two-tower network, we integrate the output of matching network as features into the multitask learning embedding layer, which increases feature expression ability and helps different tasks to extract information more accurately.
3. To our best knowledge, this is the first work that introduces mimic interaction into the MMOE-match network. It adds the communication of recalling and ranking, which makes combined training better.
4. Combined with TCM [18] knowledge, we design the statistical features such as confidence degree feature, promotion degree feature, and TF-IDF feature. The experiments show that these features bring a positive effect.
5. We show offline results of our experimental evaluation on TCM syndrome recommended data in laboratory, which demonstrates the scalability of our methods.

## 2. Related Work

Our work is based on the algorithm of artificial intelligence recommendation system, which is applied in TCM syndrome element recommendation. The first is multitask learning, and the second is a two-tower matching network, which can improve the convergence and prediction accuracy.

### 2.1. Recommendation Systems

Recommendation systems select and rank items from millions of candidates. It is a common practice that uses models with two stages: the recalling stage and the ranking stage. The recalling stage reduces the corpus size from millions to thousands, and the ranking stage estimates the CTR of item candidates and delivers top-ranked items to users. During the ranking stage, machine learning or deep learning has been widely used. Logistic regression [19] is a classic method that recommends items based on the linear structure quickly. XGBoost [20] is a gradient boosting tree learning algorithm, which is widely used in many winning solutions of machine learning competitions. With the development of deep learning, the neural network models have been successfully applied for recommendation CTR prediction. Wide-deep [21] jointly utilizes both linear memory and nonlinear generated abilities via its wide and deep architecture. Deep-FM [22] combines DNN with neural FM to capture high-order interaction automatically.

### 2.2. Multitask Learning

Multitask learning (MTL) has achieved success in many applications of deep learning, from natural language processing and speech recognition to computer vision and recommender systems. Compared with single-task learning, it can significantly improve learning efficiency and prediction accuracy via using a flexible share mechanism for different tasks. The mainstream of multitask learning is Expert-Gate pattern, Expert-NAS pattern, and Probability-Transfer pattern. The main idea of Expert-Gate pattern is to control how experts are shared or independent among tasks, including MMOE [23], DADR [24], MOSE [25], and PLE [26]. The Expert-NAS pattern can learn expert or feature information selectively and choose knowledge across all tasks in a flexible and efficient way, including SNR [27] and MSSM [14]. The Probability-Transfer pattern considers relationships in the output layer of different tasks, which can transfer information via scalar product and model sequential dependence well among rich useful representations, including ESMM [28], ESM2 [29], and AITM [16].

### 2.3. Matching Tower in Recommendation

In the recalling stage of recommendation systems, the mainstream model is two-tower match networks. YouTube-Net [3] highlights the two-step architecture in a wide range of real-world applications, which brings in deep neural networks to build user embedding for matching. Recently, graph embedding [30] models are rising to learn the representation of items and users. The recalling process is made equivalent to retrieve the nearest neighbors of users' vectors among all the items. Inspired by the matching idea, our work introduces two-tower models in building multitask learning in large-scale recommenders.

### 2.4. Application of Recommendation Algorithm in TCM Syndrome Elements

TCM diagnosis needs to predict syndrome elements, symptoms, disease name, and prescription name according to the patient's medical record. The traditional approach views it as multilabel problems, which employ natural language processing techniques such as TextCNN model. When the number of predict objects is small, the multilabel algorithm is feasible, but when the number of predict objects is large, the multilabel algorithm is not good. There are more than 60 kinds of TCM syndrome elements, more than 2000 kinds of symptoms, and more than 10,000 kinds of disease names and prescription names, so it is extremely important to explore prediction schemes beyond multilabel mode.

The e-commerce recommendation system mines the user interested commodities from massive data, and the candidate objects are much larger than TCM predict objects. We refer recommendation ranking algorithm and introduce deep learning in TCM projects. Syndrome element predict, symptom predict, name of disease predict, and name of prescription predict, each of them can be trained independently using the recommendation ranking algorithm. It also can be trained jointly and viewed as multiple tasks, which use one model to obtain multiple prediction results at the same time.

## 3. The Proposed Methods

**Figure 1:**
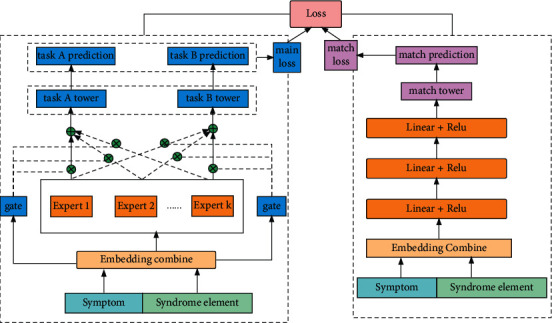
MMOE-match network.

We design a general ranking paradigm called MMOE-match, which combined multitask learning with matching network. We apply it in the laboratory TCM medical case data and achieve good performance, which is significant for the construction of TCM recommendation system. The core structure of neural network proposed by us is shown in Figure 1.

On the left side of Figure 1 is the multitask learning model, which is the main network. From bottom to top are input data layer, embedding layer, expert network, gate network, tower layer, and prediction layer.

On the right of Figure 1 is the double-tower matching network. From bottom to top are input data, embedding layer, MLP layer, match tower layer, and match prediction layer.

On the left side of Figure 1 (main network) is as follows:

  Input Data: it includes symptoms and syndrome elements. Symptoms are used to construct features, and syndrome elements are used to construct labels.
  Embedding Layer: the symptoms are text features, which are processed as ID features via word segmentation. Embedding transforms high-dimensional sparse symptom ID feature into the low-dimensional dense vector by nonlinear mapping.
  Expert Layer: it is two fully connected layers. We design eight experts, which are used to extract abstract feature information.
  Gate Layer: it is used to represent the weight of experts among different tasks.
  Tower Layer: it is two fully connected layers, and the number of towers is the same as the number of tasks.
  Prediction Layer: it is a nonlinear mapping that represents the probability of the positive sample.

On the right side of Figure 1 (auxiliary network) is as follows:

  Input Data: it is the same as the input of the main network named multitasking learning.
  Embedding Layer: the processing mode is the same as that of the main network.
  MLP Layer: it is 3 fully connected layers using linear transformation and activation function.
  Match Layer: it is three fully connected layers with L2 norm.
  Prediction Layer: it is a nonlinear mapping, which indicates the probability of matching symptoms and syndrome elements.

In Figure 1, total loss includes main network loss and auxiliary network loss. The Adam optimizer is used for gradient updating to continuously reduce the total loss, which obtains the optimal solution.

### 3.1. Improve Method 1: Multitask Learning Introducing Matching Network

We predict the syndrome elements in the TCM project, and each medical case has a real syndrome element. There are more than 60 syndrome elements in 150,000 medical case sets. The method of constructing training samples is as follows: if a medical case corresponds to the real evidence element, then it is label 1; if the example corresponds to the evidence element that does not belong to itself, then it is label 0. The recommendation system divides into two main stages, recalling and ranking. In the recall stage, the candidate items are produced from massive data and then provided to the ranking stage. Generally speaking, the recall data are large and the model is a simple two-tower model. With fewer data in the ranking stage, various complex models can be optimized. We regard syndrome recommendation as the ranking stage and use multitask learning MMOE. Since features are symptoms and syndrome elements, inspired by the user side and item side of the two-tower model in the recall stage, a matching tower is designed to assist ID feature learning better.

The original inputs are symptoms and syndrome elements, as shown as follows:

(1) $$\begin{array}{c} \text{input}=\left[X_{zz},X_{zs}\right], \end{array}$$

where *X*_*zz*_ represents TCM symptom data and *X*_*zs*_ denotes TCM syndrome element data.

The embedding layer maps the high-dimensional sparse vector to the low-dimensional dense vector space, which is expressed as follows:

(2) $$\begin{array}{c} E\left(\theta \right)=\left[e_{1},e_{2},\ldots ,e_{n}\right]=f\left(X_{zz},X_{zs}\right), \end{array}$$

where [*e*_1_, *e*_2_,…, *e*_*n*_] represents concatenating multiple vectors. The detailed processing steps of *f*(*∗*) are as follows: word embedding is performed on the words in each field, including the syndrome, tongue, moss, pulse, and syndrome elements, respectively. Then, the average is taken to obtain the vector representation of each field, and the vectors of each field are concatenated to get the vector representation of the input data.

The output of the multitask learning tower is shown as follows:

(3) $$\begin{array}{c} {\hat{y}}_{k}=t^{k}\left(f^{k}\left(E\right)\right), \end{array}$$

where *t*^*k*^(*∗*) represents the Kth tower and *f*^*k*^(*∗*) denotes the linear combination of gate network and expert network.

(4) $$\begin{array}{c} f^{k}\left(E\right)=\sum_{i=1}^{n}g^{k}{\left(E\right)}_{i}f_{i}\left(E\right), \end{array}$$

(5) $$\begin{array}{c} g^{k}\left(E\right)=\text{softmax}\left(W_{gk}E\right), \end{array}$$

where *g*^*k*^(*∗*) represents gate function, and it is a softmax activation function. *W*_*gk*_ represents the weight of a gate network.

(6) $$\begin{array}{c} f_{i}\left(E\right)=FFN\left(E\right), \end{array}$$

where *FFN*(*∗*) represents two-layer feed-forward networks, and the number of nodes is 256 and 128, respectively.

The input of the matching network is symptom feature and syndrome element feature. After multiple layers of forward propagation, the matching probability is calculated, and the formula is as follows:

(7) $$\begin{array}{c} h_{1}=Relu\left(W_{1}*\left[X_{zz},X_{zs}\right]+b_{1}\right),\\ h_{L}=Relu\left(W_{L}*h_{L-1}+b_{L}\right), \end{array}$$

where *W*_1_ and *W*_*L*_ represent the model weights. *X*_*zz*_ represents TCM symptom data, and *X*_*zs*_ denotes TCM syndrome element data. *b*_1_ and *b*_*L*_ denote the bias. *Relu*(*∗*) is a nonlinear activate function.

(8) $$\begin{array}{c} {\hat{y}}_{m}=sigmoid\left(p\left(h_{L}\right)+\left.b\right)\right), \end{array}$$

where *p*(*∗*) is a nonlinear mapping and *b* represents bias.

Matching network as the auxiliary task is used to assist the main task of syndrome element prediction. We design a matching hyperparameter *λ*_1_ weighted sum of the loss function of the main task and auxiliary task, which is expressed as follows:

(9) $$\begin{array}{c} \text{Loss}={\text{loss}}_{\text{main}}+\lambda_{1}{\text{loss}}_{\text{match}},\\ {\text{loss}}_{\text{main}}=-\frac{1}{N}\sum_{k=1}^{N}\left(y_{k}*\log\left({\hat{y}}_{k}\right)+\left(1-y_{k}\right)*\text{log}\left(1-{\hat{y}}_{k}\right)\right.,\\ {\text{loss}}_{\text{match}}=-\frac{1}{N}\sum_{k=1}^{N}\left(y_{k}*\log\left({\hat{y}}_{m}\right)+\left(1-y_{k}\right)*\text{log}\left(1-{\hat{y}}_{m}\right)\right., \end{array}$$

where *y*_*k*_ is the true label and ${\hat{y}}_{k}$ and ${\hat{y}}_{m}$ are predict values.

### 3.2. Improved Method 2: Match Network Output as Multitask Learning Feature

We try to take the output of matching network as the feature of the syndrome element main prediction task, and the combined features are as follows:

(10) $$\begin{array}{c} {\text{feature}}_{\text{all}}=\left[E,{\hat{y}}_{m}\right]. \end{array}$$

Then, we put feature_all_ into equations (2)–(6), which enrich the feature representation ability to multitask learning.

**Figure 2:**
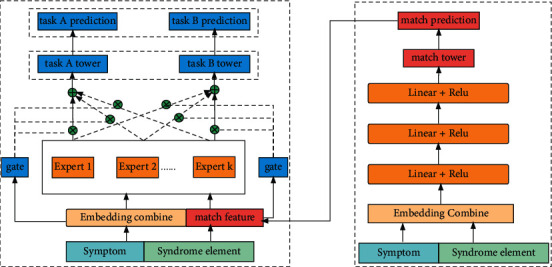
Match network prediction as MMOE feature embedding.

The model network structure diagram of improved method 2 is shown in Figure 2.

On the left side of Figure 2 is the main network called multitask learning, and on the right side is the auxiliary network called a two-tower matching. The internal structure of the main and auxiliary networks is the same as shown in Figure 1. The difference is that the output of the auxiliary network in Figure 2 is taken as a part of the features of the main network, and the prediction accuracy is improved via feature richness.

**Figure 3:**
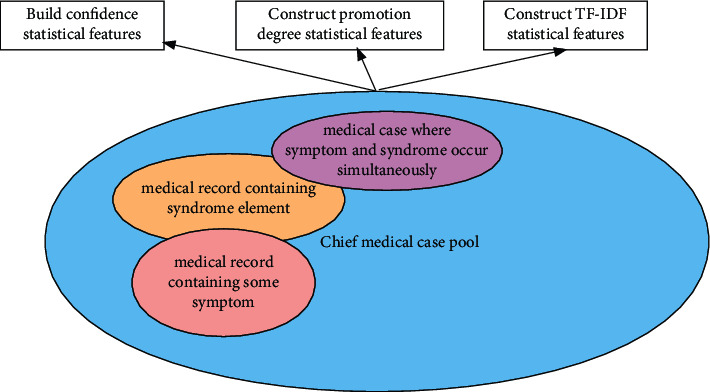
Statistical features of artificial design based on medical knowledge.

To explore the relationship between features and labels and extract abstract features, we design the cross-features of symptom and syndrome element based on TCM knowledge. The core construction logic of the cross-features will be introduced in the experiment section. The sources of statistical feature calculation are summarized in Figure 3.

The details of the experiment are given in Section 4, and from the experiments, we can know that

1. Comparing improved method 1 and method 2 with the base multitask model, the experimental effects are all better than MMOE. It indicates that matching network can learn feature cross-information. Feature engineering is abstracted into models, which can help us save the cost of manual design. The matching network results are combined as MMOE features, which brings richer feature knowledge.
2. Comparing improved method 2 with our artificial design features, we know that there are different ways of adding new features. The fact denotes that the gain brings by well-designed features is also very significant. The man-made syndrome elements and symptom statistical features need to be familiar with the business knowledge, and the knowledge of TCM should be combined to guide model learning.

Conclusion: in the early stage of business, when we do not have a lot of feature experience, the idea of model matching is a worthy direction to try. In the later stage of business, experience guides feature design better.

### 3.3. Improved Method 3: Mimic Interaction Mechanism between Recalling and Ranking

The core of recalling and ranking is a two-tower matching network, and multitask learning has been studied long before. In fact, it is our innovation to combine recalling and ranking in one model. Match network to model is not a new problem; however, how to better interact with match network and ranking model is a worth exploring problem. To provide better interaction between MMOE and match network, we introduce a mimic interaction mechanism, which can assist ID feature learning. The interaction mechanism is represented by *a*_*u*_,  *a*_*v*_ vector and *p*_*u*_,  *p*_*v*_ operator. The detailed mathematical expression of improvement is as follows.

**Figure 4:**
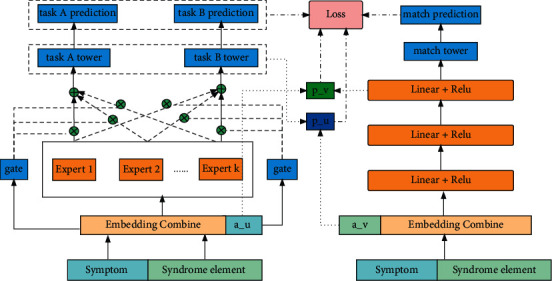
MMOE-match-mimic network.

On the left of Figure 4 is the main network multitask learning, and on the right side is the two-tower matching auxiliary network. The internal structure of the main network and the auxiliary network is the same as in Figures 1 and2. The innovation is that *a*_*u*_,  *a*_*v*_ vectors and *p*_*u*_,  *p*_*v*_ operators are added in Figure 4 to make the recalling and ranking better interactive.

(11) $$\begin{array}{c} c_{u}=\left[e_{1}\left|\left|e_{2}\right|\right|\ldots \left|\left|e_{n}\right|\right|a_{u}\right],\\ c_{v}=\left[e_{1}\left|\left|e_{2}\right|\right|\ldots \left|\left|e_{n}\right|\right|a_{v}\right], \end{array}$$

where || is the vector concatenation operation. The vectors *c*_*u*_ and *c*_*v*_ not only mean information about the current symptom and syndrome element but also contain information about historical positive interactions through *a*_*u*_ and *a*_*v*_.

(12) $$\begin{array}{c} m_{1}=Relu\left(W_{1}*c+b_{1}\right),\\ m_{L}=Relu\left(W_{L}*m_{L-1}+b_{L}\right),\\ p=L2Norm\left(m_{L}\right), \end{array}$$

where *c* means *c*_*u*_ and *c*_*v*_ and *p* means *p*_*u*_ and *p*_*v*_; *W*_*L*_ and *b*_*L*_ are the weight matrix and bias vector for the Lth layer. *p*_*u*_ and *p*_*v*_, the output vectors of the L2 normalization layer, represent the MMOE embedding and match-net embedding, respectively.

(13) $$\begin{array}{c} {\text{loss}}_{u}=\frac{1}{N}\sum \left[ya_{u}+\left(1-y\right)p_{v}-p_{v}\right], \end{array}$$

(14) $$\begin{array}{c} {\text{loss}}_{v}=\frac{1}{N}\sum \left[ya_{v}+\left(1-y\right)p_{u}-p_{u}\right], \end{array}$$

(15) $$\begin{array}{c} \text{Loss}={\text{loss}}_{\text{main}}+\lambda_{1}{\text{loss}}_{\text{match}}+\lambda_{u}{\text{loss}}_{u}+\lambda_{v}{\text{loss}}_{v}, \end{array}$$

where *λ*_1_, *λ*_*u*_, *λ*_*v*_ are tunable parameters.

## 4. Experiment

### 4.1. Data Sets

Our laboratory has been conducting research on TCM based on big data for decades and has undertaken and completed dozens of TCM projects and accumulated a large number of medical records of real patients. Up to now, there are a total of 480,000 medical cases, and we have full scientific research rights. The data set used in this research is derived from it. The diagnosis and treatment of medical cases are the generations of famous old Chinese medicine doctors, such as Pei-Sheng Li and Ren He. Pei-Sheng Li was the first batch of old Chinese academic experience heir guidance teacher, and his representative works include “Treatise on febrile diseases” and “Annotation on Koch Febrile Diseases.” Ren He was the first “national physician master” honorary title winner, and his popular works include “Synopsis of the Golden Chamber,” “Synopsis of the Golden Chamber,” and “Synopsis of the Golden Chamber.” Medical records include five aspects of information: patient's personal information, such as gender and age; environmental information at the time of medical treatment, such as the date of medical treatment and solar terms; patient's disease description information, such as engraving and tongue; patient's medical history, such as genetic disease and past history; and doctors' analysis of patients' diseases, diagnosis, and treatment information during diagnosis and treatment, such as syndrome and treatment principles and methods. Although there is no standard specification for TCM, the description of our medical records is taken from standard processing, which uses years of technical experience accumulated in the laboratory. All aspects of the information in the medical case are relatively standardized. Fields that are abstract and difficult to predict by models are also processed according to national standards and specifications, such as syndromes. Syndromes are separated by fine granularity according to TCM syndromes, so that the information of etiology, disease location, and disease nature can be more clearly displayed.

The purpose of this study is integrating the way of patients' diagnostic thinking and treatment into the deep learning model based on the idea of recommendation. We hope that the model can imitate the diagnosis and treatment methods of famous TCM doctor, which can carry out the diagnosis and treatment of diseases better. The first step of diagnosis and treatment is to analyse the location and severity of the disease based on the patient's disease information, namely the syndrome element prediction. It is also the stage of diagnosis and treatment in our experiment. We screen 150,000 medical cases from the data set and randomly select 130,000 of them as the training set and the remaining 20,000 as the test set. Then, we extract the syndrome, tongue, moss, and pulse information from medical cases to generate the syndrome element information data set. We regard the syndrome as the recommendation target.  Table 1 shows examples of medical cases. For privacy reasons, only the fields of the syndrome, tongue, moss, pulse, and syndrome elements used in this study are shown here. It is worth noting that, when generating the data set, a medical case is equivalent to a user recommended sample in e-commerce, and the certificate is equivalent to the goods to be recommended. Finally, the number of training samples is 8.06 million, and the number of test samples is 1.24 million.

In addition, based on previous experimental experience, we design some statistical features, which also play a vital role in the prediction of the syndrome element. The statistical computing methods include confidence degree feature, promotion degree feature, and TF-IDF feature. Taking the statistics of the underlying symptoms and syndrome elements as an example, the calculation methods are as follows:

(16) $$\begin{array}{c} F_{1}=\frac{N_{zz}\cap N_{zs}}{T_{zz}}, \end{array}$$

(17) $$\begin{array}{c} F_{2}=\frac{N_{zz}\cap N_{zs}}{T_{zs}}, \end{array}$$

where *F*_1_ and *F*_2_ denote the two types of confidence degree feature computing methods. *N*_*zz*_ denotes the number of symptoms that occur, and *N*_*zs*_ denotes the number of syndrome elements that occur. *T*_*zz*_ means the number of medical cases containing the current symptoms, and *T*_*zs*_ means the number of medical cases containing the current syndrome elements.

(18) $$\begin{array}{c} L=\frac{F_{2}}{T_{zs}/T_{\text{total}}}*1e5, \end{array}$$

where *L* denotes the promotion degree feature and *T*_total_ means the number of total medical cases.

(19) $$\begin{array}{c} \text{TF}=\frac{N_{zz}\cap N_{zs}}{S_{zz}}, \end{array}$$

where *S*_*zz*_ denotes the number of current symptoms that appear with whole syndrome elements at the same time.

(20) $$\begin{array}{c} \text{IDF}=\text{log}\frac{Y_{zz}}{H_{zs}+1}, \end{array}$$

where *Y*_*zz*_ denotes the number of whole symptoms and *H*_*zs*_ means the number of current syndrome element that appears with other syndrome elements at the same time.

(21) $$\begin{array}{c} \text{TF}-\text{IDF}=\text{TF}\times \text{IDF.} \end{array}$$

On the basis of the above statistical method, in the training set, we do statistics on the engraving symptoms, tongue, moss, pulse, and syndrome element separately. Then, we calculate the statistical information of each medicinal case via statistical results. In each sample, the engraving symptoms, tongue, moss, and pulses containing words are not a fixed length, so we take the further processing. The processing methods include summation, averaging, and padding. The padding rule is defined: maximum length of engraving symptom is 7; if the length is insufficient, it will be filled −1; and if the length is redundant, it will be truncated. The retention length of tongue, moss, and pulse is 1, which means the average of the corresponding statistical information of each medical case. Taking one of the samples as an example, the statistical information generation process is shown as follows.

Firstly, we combine the sample information and statistical information to obtain the statistical values of the engraving symptoms, tongue, moss, pulse, and syndrome elements. Secondly, the statistical values were calculated by summing, averaging, and padding.

The sample information is shown in Table 2.

The corresponding statistical values of samples are shown in Table 3.

Here, *F*_1_ and *F*_1_ denote two types of confidence degree features, and *L* means the promotion degree feature.

The sample statistical features are shown in Table 4.

### 4.2. Compare Models

TextCNN [31]: TextCNN is a multilabel framework, which uses a convolution neural network to construct the model structure.

MLP [32]: multilayer perceptron is a base structure of deep learning and is widely applied in the recommendation system, which we use as our baseline.

MMOE [23]: the MMOE with Expert-Bottom pattern can integrate experts by multiple gates in the gate weights.

Two-Tower Model [7]: a deep two-tower neural network is based on the recommendation method proposed by YouTube. Vectors of items and users are concatenated and fed into a multilayer feed-forward neural network.

MMOE-Match-Mimic: this is our proposed model, which combines a match network and multitask learning network together.

### 4.3. Parameter Setting

All the above neural network models are realized based on the TensorFlow framework. We use a GPU (Tesla V100-PCIE-32 GB) server to train and test each model. To make the models comparable, we maintain consistency in data sets, hyperparameters, and model structures as much as possible. TextCNN is implemented based on the idea of multilabel classification, and data sets need to be constructed independently, so this model has the biggest difference from other models. It should be emphasized that the medical case fields used in the training and test sets are uniform for all models. TextCNN employs the Adam optimizer to tune the model with a learning rate of 0.001. Each batch contained 32 medical cases. The model uses the Albert pretraining model to embed the input, and convolution kernels with lengths of 4, 5, and 6 are, respectively, used for one-dimensional convolution. Finally, the outputs of each convolution layer are spliced through the full-connection layer for multilabel prediction. The activation function of the full-connection layer is sigmoid. In other networks, multilabel problems are converted into N dichotomous problems for processing, so there is a good uniformity in the setting of hyperparameters. The Adam algorithm is used to update model parameters with a learning rate of 0.0005. Each batch contains 5000 samples. The word table of the embedding layer is randomly initialized based on normal distribution and updated with the training of the model. MLP network is a three-layer fully connected network, and the nodes of each layer are 256, 128, and 64, respectively. MMOE contains 8 expert modules, each of which is a two-layer fully connected network with 256 and 128 nodes, and the tower layer is a one-layer fully connected network with 64 nodes. The structure of the two towers in the match network is consistent with MLP. The MMOE module and two-tower module in the MMOE-two-tower network are consistent with the settings of the MMOE and two-tower model, respectively.

All the model parameters are randomly initialized based on truncated normal distribution, and there is a certain fluctuation in the evaluation. To prevent the interference of index fluctuation on the results, the model is trained 5 times in each experiment, and the average value of the evaluation index of each model is taken.

### 4.4. Evaluate Metrics

There are 5 evaluation indicators in our experiments, and they are area under ROC curve (AUC), RelaImpr, Hits@10, MeanRank, and MRR.

#### 4.4.1. AUC [33]

AUC measures the performance of ranking model with a predicted value in the test data set. We define AUC as follows, and the higher the better:

(22) $$\begin{array}{c} \text{AUC}=\frac{1}{\left|T^{+}\right|\left|T^{-}\right|}\sum_{x^{+}\in T^{+}}\sum_{x^{-}\in T^{-}}\left(f\left(x^{+}\right)>f\left(x^{-}\right)\right), \end{array}$$

where *T*^+^ denotes the positive and negative samples, |T ^ + | and |T ^ - | are the number of positive and negative samples, *f*(*x*) is the predict function, and *I*(*x*) is the indicator function.

#### 4.4.2. RelaImpr

RelaImpr represents the relative improvement effect, the higher the better, and we define it as follows (23):

(23) $$\begin{array}{c} RelaImpr=\left(\frac{\text{AUC}\left(\text{new model}\right)-0.5}{\text{AUC}\left(\text{base}\right)-0.5}-1\right)*100\%. \end{array}$$

#### 4.4.3. Hits@10

In the label set of a medical record, Hits@10 denotes the probability of the first 10 hits in the output, the higher the better, and we define it as formula (24):

(24) $$\begin{array}{c} Hits@10=\frac{\text{label}\cap pred\_10}{\text{numbers of label}}, \end{array}$$

where pred_10 means the top 10 predict syndrome elements.

#### 4.4.4. MeanRank

MeanRank is used to measure the likelihood that the model will incorporate errors, the smaller the better, and we define it as (25):

(25) $$\begin{array}{c} \text{MeanRank}=\left\{\begin{array}{cc} \frac{y}{s}, & \;i<5,\\ \frac{y+i-3}{s}, & 5\leq i\leq 10,\\ \frac{11-s}{s}, & \text{other,} \end{array}\right. \end{array}$$

where *y* denotes the overlap number of the corresponding syndromes of the real medical record with the top 10 predict syndromes, *s* denotes the number of real syndrome elements, and *i* is the ranking position of predict syndrome elements.

#### 4.4.5. MRR

MRR indicates the generalization ability and robustness of the model, the larger the better, and we define it as follows (26):

(26) $$\begin{array}{c} \text{MRR}=\frac{1}{\text{MeanRank}}. \end{array}$$

### 4.5. Offline Results

In this section, we compare our proposed method with several benchmark models. The TextCNN is a common model to solve the problem of multilabel classification. MLP is a classical neural network, which is widely used as baseline. MMOE is the representation of multitask learning, which is the model we proposed based on, so it is natural to compare with it. It can be seen from Table 5 that our proposed model has advantage in all indicators. Hits@10 is the indicator we pay attention to, and our model has obvious improvement in this indicator. It is worth noting that MMOE does not perform well in the experiment, and its AUC is even lower than MLP, because MMOE is a multitask model. However, due to the limitation of data set, our experiment is a single-task scenario, and MMOE could not give full play to its advantages. The model we proposed is based on MMOE, which can better demonstrate the superiority of match network in this scenario.

### 4.6. Ablation Experiment

1. Our proposed model extracts the features between symptoms and syndrome elements via the match module and acts as the match output on loss function to assist network updating. To verify the effects, we separate them and conduct experiments with MMOE and match model, respectively. The experimental results are shown in Table 6. It can be seen that using a match module to assist loss function updating and employing match-net for automatic feature extraction can both play a certain effect, and the combination of the two achieves more obvious effects.
2. We construct a series of features via the method in 4.1 and add these features to the MMOE for training, respectively. We compare them with our model, and the experimental results are shown in Table 7, as is shown that most of the carefully designed features are better than the automatically extracted features by the model. However, there are also some cases, such as the promotion degree feature, which reduces the model prediction effect. Our model adopts a match module to automatically extract features, which shows certain well effects. When a new business needs to be launched quickly or the domain knowledge is insufficient, it is difficult to construct artificial features. Under this circumstance, our proposed model does not require any prior knowledge, and features are completely learned automatically. It not only improves the speed of business going online, but also reduces the dependence of features on domain knowledge.

## 5. Conclusions

In this study, we propose MMOE-match-mimic network to model the relationship between symptoms and syndrome elements. The proposed match network module combines the multigate mixture-of-experts in the loss function. In this way, it can learn more information of different ID embeddings to improve the performance of multi-task learning. To our best knowledge, this is the first work that introduces mimic interaction into the MMOE-match network. The work adds the communication of recalling and ranking, which makes combined training better. The mimic mechanism aims to model the information interaction between MMOE-match networks and produces better symptom representations for TCM data. We conduct extensive experiments and obtain 0.705% increase in the AUC, which demonstrates the effectiveness of MMOE-match-mimic for TCM symptom recommender. Moreover, MMOE-match-mimic has been successfully deployed to serve the laboratory TCM health diagnostic system, which shows significant improvements compared with state-of-the-art baseline models.

## Figures and Tables

**Table 1:** Examples of medical case.

Syndrome	Tongue	Moss	Pulse	Syndrome elements
Abdominal distension, spontaneous perspiration, proteinuria	Red tongue, plumpness of tongue	White coating	Stringy pulse, rapid pulse	Kidney, deficiency, water, flooding
Edema, proteinuria, abdominal distension, soreness of waist	Red tongue, plumpness of tongue	White coating	Rapid pulse, thready pulse	Spleen, stomach Qi, deficiency, phlegm, block, Qi, retardation
Increased eating with rapid hungering, choking sensation in chest, abdominal distension, dry mouth, pruritus vulvae	Big tongue	White coating, greasy coating	Deep pulse, smooth pulse	Spleen, deficiency, wetness, sheng
Pain in the stomach, burning sensation in the stomach, belching, nausea, dry mouth, vexation, deficiency of food	Reddish tongue	Little coating	Thready pulse	Cold, hot, interties
Dementia, dysphoria, feeling uncertain, lethargy, degraded memory, eyes are unable to open, numbness, glazed eyes, soliloquy	Dark red tongue	Yellow coating, greasy coating	Smooth pulse	Phlegm, faint

**Table 2:** Sample information.

Engraving symptoms	Tongue	Moss	Pulse	Syndrome elements
Nausea, vomiting,	Plumpness of tongue	Yellow coating, greasy coating	Thready pulse	Wetness
Abdominal pain, flatus, weakness				

**Table 3:** Corresponding statistical values of samples.

	Engraving symptoms	Tongue	Moss	Pulse
*F*_1_	0.0821, 0.0698, 0.0850, 0.0855, 0.0575	0.0653	0.0840, 0.1049	0.0985
*F*_2_	0.3343, 0.2703, 0.3099, 0.3379, 0.2497	0.2805	0.3415, 0.4384	0.3960
*L*	1.0938, 0.8844, 1.0139, 1.1056, 0.8170	0.9178	1.1172, 1.4345	1.2958
TF-IDF	0.1823, 0.1550, 0.1888, 0.1900, 0.1277	0.0910	0.0943, 0.1179	0.1366

**Table 4:** Sample statistical features.

	Sum	Average	Padding
*F*_1_	0.7327	0.0814	0.0821, 0.0698, 0.0850, 0.0855, 0.0575, −1, −1, 0.0653, 0.0945, 0.0985
*F*_2_	2.9585	0.3287	0.3343, 0.2703, 0.3099, 0.3379, 0.2497, −1, −1, 0.2805, 0.3899, 0.3960
*L*	9.6800	1.0756	1.0938, 0.8844, 1.0139, 1.1056, 0.8170, −1, −1, 0.9178, 1.2759, 1.2958
TF-IDF	1.2835	0.1426	0.1823, 0.1550, 0.1888, 0.1900, 0.1277, −1, −1, 0.0910, 0.1061, 0.1366

**Table 5:** Evaluation index of different models.

Model	Hits@10	MeanRank	MRR	AUC	RelaImpr
MLP	0.76644	3.00478	0.33280	0.90844	—
TextCNN	0.77080	2.95720	0.33812	0.90991	+0.360%
MMOE	0.76824	2.98508	0.33500	0.90780	−0.157%
Our model	0.77281	2.95292	0.33894	0.91132	+0.705%

**Table 6:** Comparison of the match module effect.

Model	Hits@10	MeanRank	MRR	AUC
MMOE	0.76824	2.98508	0.33500	0.90738
Only match-net	0.75623	2.99645	0.33372	0.89786
MMOE-match-auxiliary-loss	0.77146	2.95178	0.33878	0.90964
MMOE-match as features	0.77134	2.95752	0.33812	0.90986
MMOE-match-mimic	0.77185	2.95638	0.33846	0.90571
Our model	0.77281	2.95292	0.33894	0.91132

**Table 7:** Match-net extract features compared with designed features.

Model	Hits@10	MeanRank	MRR	AUC
MMOE	0.76824	2.98508	0.33500	0.90738
Only match-net	0.75623	2.99645	0.33372	0.89786
Zxd1:avg|sum	0.77932	2.90490	0.34426	0.90702
tfidf:avg|sum	0.77980	2.90894	0.34378	0.90550
Zxd2:avg|sum	0.77624	2.92736	0.34160	0.90466
tsd:avg|sum	0.76554	2.99182	0.33426	0.90694
Zxd1:padding	0.77514	2.93590	0.34062	0.90822
tfidf:padding	0.77538	2.93682	0.34048	0.90664
Zxd2:padding	0.77514	2.93754	0.34042	0.90436
tsd:padding	0.76740	2.97964	0.33562	0.90834
Our model	0.77281	2.95292	0.33894	0.91132

## Data Availability

The data set comes from medical records of 480,000 real patients collected in the laboratory.
